# Testing the validity and responsiveness of a new cancer-specific health utility measure (FACT-8D) in relapsed/refractory mantle cell lymphoma, and comparison to EQ-5D-5L

**DOI:** 10.1186/s41687-020-0185-3

**Published:** 2020-03-27

**Authors:** Michael Herdman, Cicely Kerr, Marco Pavesi, Jamie Garside, Andrew Lloyd, Patricia Cubi-Molla, Nancy Devlin

**Affiliations:** 1 Office of Health Economics, Southside, 7th Floor, 105 Victoria Street, London, SW1E 6QT UK; 2 Janssen-Cilag Ltd, High Wycombe, UK; 3 Data Management Centre, European Foundation for the Study of Chronic Liver Failure (EF-CLIF), Barcelona, Spain; 4 Acaster Lloyd Consulting Ltd, London, UK

**Keywords:** Cancer, Quality of life, Utilities, Condition-specific non-preference-based measures, Generic preference-based measures

## Abstract

**Background:**

The FACT-8D is a new cancer-specific, preference-based measure (PBM) of health, derived from the Functional Assessment of Cancer Therapy – General (FACT-G) questionnaire. The FACT-8D’s measurement properties have not been tested to date. We assessed it’s validity and responsiveness in relapsed/refractory mantle cell lymphoma (RR MCL) and compared the results to the EQ-5D-5L.

**Methods:**

Blinded analysis of pooled data from a phase 3 clinical trial. FACT-8D baseline and follow-up data (weeks 4, 7, 16, 31) were scored using Australian preference weights, the first available value-set. Convergent validity was assessed by estimating baseline correlations with the FACT-Lym total score, Trial Outcome Index (TOI), FACT-Lym lymphoma-specific sub-scale (LymS), EQ-5D Visual Analog Scale (VAS), and haemoglobin (HgB). Relevant clinical variables were used to categorise patients to test known groups’ validity and responsiveness was investigated using data from baseline (*n* = 250) and week 31 (*n* = 130). Results were compared with EQ-5D-5L, scored using the UK 3L crosswalk and the 5L England value-sets.

**Results:**

The FACT-8D showed good convergent validity and responsiveness; baseline Pearson correlation coefficients between FACT-8D Index scores and other PRO measures were moderate to very strong (range: 0.49 for the EQ-VAS to 0.79 for FACT TOI) and the size of the change in FACT-8D Index scores at week 31 differed significantly (*p* < 0.005) between patients categorised as improved, worsened or stable using the FACT-Lym total score, LymS, and HgB. However, when assessing known groups’ validity, FACT-8D failed to discriminate between patients categorised by health status on four of the seven variables analysed. Overall, FACT-8D and EQ-5D-5L performed similarly, although EQ-5D-5L showed better known groups’ validity.

**Conclusions:**

This is the first investigation into the psychometric properties of the FACT-8D. In this RR MCL trial dataset, it showed good convergent validity and responsiveness, but poorer known groups’ validity, and EQ-5D performed as well or better on the tests conducted. The FACT-8D may offer an alternative method to generate utilities for the cost-effectiveness analysis of cancer treatments but needs further testing in other types of cancer patients. Evaluation of utility gains may have been limited by high baseline performance status in this RR MCL trial sample.

## Introduction

Generic preference-based measures (PBMs) of patient-reported health, particularly the EQ-5D, form the cornerstone of health-related quality of life (HRQOL) measurement for economic evaluation in the context of health technology assessment [1, 2]. The EQ-5D was designed as a simple instrument which could be used to measure, compare and value health status in both the general population and across a wide range of health conditions [3].

However, in recent years, several condition-specific PBMs have been developed which could also be used in economic evaluations, including instruments designed to derive health utilities in cancer [4], overactive bladder [5], epilepsy [6], or asthma [7]. As condition-specific questionnaires assess aspects of HRQOL which are of particular relevance to a given patient population, they might be expected to be more valid and sensitive in those patients than generic measures. In this regard, the EQ-5D has occasionally been criticised as insensitive and unresponsive to differences and changes in health status in some conditions, including cancer, although often without empirical evidence to support such claims [8]. For example, one area in which the EQ-5D-5L has been challenged is in the measurement of fatigue [8]. Analysis of a large cancer dataset in which the EQ-5D-3L was compared to the EORTC-8D, a cancer-specific PBM, suggested that the EQ-5D-3L may fail to pick up impairments in fatigue and sleep disturbance, although both measures were found to be responsive and sensitive to disease characteristics [9].

One recently developed cancer-specific PBM is the FACT-8D [10], which was derived from the Functional Assessment of Cancer Therapy – General (FACT-G) questionnaire, a widely used measure of HRQOL in cancer [11]. The FACT-G is part of the Functional Assessment of Chronic Illness Therapy (FACIT) Measurement System, a set of health-related quality of life (HRQOL) questionnaires intended to assess and facilitate the management of chronic illness. The FACT-G is the system’s generic core measure and was the first to be developed. The current version contains 27 items measuring HRQOL in four domains: Physical Well-Being, Social/Family Well-Being, Emotional Well-Being, and Functional Well-Being. Extensions of the instrument have been developed for use in specific forms of cancer, including one for use in patients with non-Hodgkin’s lymphoma, called the FACT-Lym [12]. The eight items composing the FACT-8D are all drawn from the FACT-G and none are specific to lymphoma patients.

To date, the measurement properties of the FACT-8D have not been tested or compared to those of a popular generic PBM such as EQ-5D. EQ-5D and the FACT-Lym questionnaire, from which the FACT-8D can be scored, were both included in a recent clinical trial of ibrutinib versus temsirolimus [13] thereby providing an opportunity to compare the performance of the two instruments in patients with relapsed/refractory mantle cell lymphoma (RR MCL). MCL is a rare and incurable B-cell lymphoma which accounts for 6–8% of all non-Hodgkin lymphomas. It has an annual incidence of 0∙4 per 100,000 persons in the USA and Europe. The condition is most common in men over 60 years and generally presents as late-stage disease. Patients with relapsed disease respond poorly to chemotherapy and progress rapidly, resulting in a median overall survival of 1–2 years. Few treatments are available for RR MCL, though recent studies have shown promising results for ibrutinib, a first-in-class, covalently binding inhibitor of Bruton’s tyrosine kinase [13]. Relative to other types of lymphoma which are mostly indolent, patients with MCL who have acquired genetic mutations have aggressive disease and shortened life expectancy, more similar to metastatic solid tumours. Other MCL patients may have a more indolent form of disease but with each additional line of therapy the quality and duration of response tends to diminish [14].

The primary objective of the present study was to test the validity and responsiveness of the cancer-specific FACT-8D in a population of RR MCL patients. A secondary objective was to compare the results with those for EQ-5D-5L.

## Methods

### Study design and population

Data for this analysis are from the RAY trial, a randomized, controlled, open-label, multicenter Phase 3 study of ibrutinib (*n* = 139) versus temsirolimus (*n* = 141) conducted in 21 countries [13]. To be included in the RAY trial patients had to meet the following inclusion criteria: have received at least one previous rituximab-containing chemotherapy regimen, have documented relapse or disease progression after the last anti-mantle-cell lymphoma treatment, have measurable disease by Revised Response Criteria for Malignant Lymphoma [15], and an Eastern Cooperative Oncology Group (ECOG) performance status of 0 or 1 [16]. Treatment was administered orally once a day on continuous cycles (ibrutinib) or intravenously on days 1, 8 and 15 of each 21-day cycle (temsirolimus). Disease progression was assessed by an independent review committee using the revised International Working Group Criteria for non-Hodgkin’s lymphoma [17] and clinical cutoff was defined as the time at which approximately 178 progression-free survival events had been observed.

### Patient reported outcomes

Patient reported outcomes (PROs) in the RAY trial were assessed using the FACT-Lym and EQ-5D-5L, administered on day 1 of every treatment cycle during the first 6 months, then every 9 weeks up to 15 months after the first dose of the study drug. Beyond that point, PROs were collected every 24 weeks until disease progression, death, clinical cutoff (FACT-Lym) or study end (EQ-5D-5L), whichever came first. Instruments were administered at the beginning of clinic visits prior to any procedures or physician interventions. The main PRO methods and results are presented in Hess et al. [18].

### FACT-8D

The FACT-8D was developed to contribute utility weights for cost-effectiveness analysis in cancer and was derived from secondary analysis of FACT-G results from 17 pooled data sets, which included 6912 patients encompassing 14 primary cancer sites [19]. Items were selected based on a series of psychometric analyses, including assessment of response distribution, confirmatory factor analysis, Rasch analysis, sensitivity to clinical features, and responsiveness. A patient survey was also performed to assess the relative importance of items within each domain.

The items in the FACT-8D cover 8 dimensions of health (pain, fatigue, nausea, problems sleeping, problems doing work, problems with support from family/friends, sadness, worries about health) with 5 levels of severity in each dimension (None, A little bit, Some, Quite a bit, Very much) over the past 7 days. The instrument generates (5^8^ =) 390,625 health states.

To date, societal valuation of the FACT-8D has only been conducted in Australia [20]. Valuations were elicited using a Discrete Choice Experiment (DCE) from a panel of individuals (*n* = 1737) drawn from the general population. States were combined with duration. Utility decrements were derived for each level of the eight dimensions and coefficients corresponding to each level in each dimension. Index scores for FACT-8D health states range from − 0.5 to 1.0.

In the present study, FACT-8D scores were derived from responses on the FACT-G, which is incorporated into the FACT-Lym. The FACT-Lym consists of the four Functional Assessment of Chronic illness Therapy - General (FACT-G) subscales (physical, social, functional, and emotional well-being) and a 15-item lymphoma-specific additional concerns subscale (LymS). Two summary scores can be calculated: the FACT-Lym total score (FACT-G + LymS) and the FACT-Lym trial outcome index (TOI) score (physical well-being + functional well-being + LymS), with higher scores representing better outcomes.

### EQ-5D-5L

The EQ-5D-5L [5] measures health status in five dimensions (mobility, self-care, usual activities, pain/discomfort, and anxiety/depression) each with five levels of severity (no problems, slight problems, moderate problems, severe problems and either extreme problems or unable to perform activity). Respondents describe their health status ‘today’ by selecting one statement in each dimension.

Societal preference weights generated using trade-off techniques are available in the form of a single index value for each of the 3125 possible health states derived from the descriptive system (EQ-5D-5L Index). We used UK and English values to assign preference weights to EQ-5D-5L health states in the current study, the first via the use of a crosswalk algorithm developed to map EQ-5D-3L values to the 5L version [21], and the second by using values elicited directly for EQ-5D-5L health states using time trade-off (TTO) and DCE in a representative sample of the general population of England [22]. Values range from − 0.594 to 1 in the crosswalk set and from − 0.285 to 1 in the England value set (EVS). For the present study, analyses involving EQ-5D-5L were performed using both the crosswalk value set and the EVS to be able to compare results using the two scoring systems [23].

The EQ-5D-5L also includes a ‘0’ (worst imaginable health) to ‘100’ (best imaginable health) visual analogue scale (EQ VAS) on which respondents rate their overall health.

### Analysis

Given that between-arm comparisons were not relevant for the study objectives, all analyses were performed on pooled trial data and the analysts blinded to treatment arm. Due to a substantial drop-out rate (complete EQ-5D and FACT-8D data was available from 250 patients at baseline, 130 patients [51%] at 31 weeks, and 87 [34%] at week 58), we restricted analysis to data collected up to week 31 to ensure sufficient numbers of patients for all analysis. Patients who died were considered missing rather than being given a utility value of 0. Any differences in sample size with earlier studies reporting results from the RAY trial [13, 18] are due to the fact that our analyses were performed only on patients with available PRO data.

All analyses were carried out in SAS version 9.4 and results were considered statistically significant at *p* < 0.05.

### Descriptive analysis

Means and standard deviations (SD) or absolute numbers and proportions were used to describe responses by dimension and for EQ-5D-5L and FACT-8D Index scores at baseline and at 4, 7, 16, and 31 weeks.

Ceiling and floor effects were calculated for the descriptive systems of the two instruments as the n (%) of patients reporting best (ceiling effect) or worst health state (floor effect).

### Convergent validity

Convergent validity was assessed by calculating the correlation between the two preference-based measures (FACT-8D and EQ-5D-5L) and the FACT-Lym total score, the TOI, LymS, EQ-5D VAS, and haemoglobin levels (HgB), which was used as an indirect indicator of fatigue. We hypothesised that FACT-8D Index scores would show strong correlation with the LymS (and obviously with the FACT-Lym total score and TOI given the shared items), moderate correlations with the EQ VAS, and weak or no correlation with HgB (continuous values). A similar pattern of correlations was expected for EQ-5D-5L, though with a potentially weaker correlation with the FACT-Lym scores, due to lack of shared content and a generic versus disease-specific perspective. It was expected that EQ-5D-5L would show a weaker correlation with HgB than the FACT-8D, given that the latter includes a fatigue domain and EQ-5D-5L does not. Pearson’s correlation coefficient was used to calculate correlations between continuous measures and Spearman’s rank correlation was used when at least one of the variables was categorical. Correlations were classed as: non-existent or weak (0–0.2), moderate (0.2–0.5), strong or very strong (> 0.5) [24].

### Known groups’ validity

Mean (SD) FACT-8D and EQ-5D-5L Index scores were estimated and compared for groups classified according to the following variables at baseline: presence of lymphoma symptoms, ECOG performance status, simplified Mantle Cell Lymphoma International Prognostic Index (MIPI) score with patients classified as low, medium, and high risk [25], haemoglobin (HgB) levels (categorised dichotomously: < > 120 g/l for women and 130 g/l for men [26]), and number of previous lines of therapy. ECOG performance status is assigned by the attending clinician with patients being classified in one of five categories: 0 (fully active, no performance restriction); 1 (restricted in physically strenuous activity but ambulatory, able to carry out work of a light or sedentary nature); 2 (ambulatory and capable of all self-care but unable to carry out any work activities. Up and about more than 50% of waking hours); 3 (capable of only limited self-care, confined to bed or chair more than 50% of waking hours); 4 (Completely disabled, cannot carry out any self-care. Totally confined to bed or chair) or 5 (dead) [16].

Between-groups comparisons were carried out using ANCOVA models with adjustment for potential confounders. In all models, potential confounders included were age, gender, ECOG status, MIPI, and prior lines of therapy except when ECOG status or MIPI were the dependent variables, in which case they were not included as confounders as well.

### Responsiveness

Responsiveness was assessed by analysing the extent to which the FACT-8D and EQ-5D-5L reflected change on the following variables: patients showing deterioration vs no change vs improvement on the FACT-Lym total score and the LymS, using previously defined minimal important difference (MID) thresholds of 6.5 points for the FACT-Lym and 5 points for the LymS [27, 28]; change in ECOG status; and change in HgB. Effect sizes, calculated using Cohen’s d, were used to show the magnitude of change and categorised as small (0.2), medium (0.5), or large (0.8 or over) [24]. For this analysis, data used were from the baseline (*n* = 250) and 31 week (*n* = 130) visits.

Responsiveness was also assessed by analysing the correlation between changes on the PBMs and changes on the FACT-Lym total score, TOI, LymS, EQ VAS, and HgB, using Pearson’s correlation coefficient.

### EQ-5D-5L and fatigue

Given the importance of fatigue in this population, cross-sectional exploratory analysis was also conducted to assess the sensitivity of the EQ-5D-5L to fatigue. Cross-walk and EVS utilities were calculated by response level on the FACT-G/FACT-8D and LymS ‘lack of energy’ and ‘tiring easily’ items.

### Analysis by ECOG status

Only patients with ECOG performance status 0 or 1 were included in the RAY trial and, of those, 47.9% were classed as ECOG0 (fully active, able to carry on all pre-disease performance without restriction). Real-world data suggests this may not represent relapsed refractory patients in UK clinical practice [29]. We therefore included an exploratory analysis to investigate differences in utility between patients who were ECOG0 at baseline and those who were classified as ECOG1. We analysed differences between the two groups on the FACT-8D and EQ-5D-5L dimensions at baseline and compared change over time using mean Index scores for all available patients at each visit.

## Results

### Sample characteristics at baseline

Mean (SD) age of the analysis sample at baseline was 66.7 years and 73.6% of the sample were male (see Table 1). The mean (SD) baseline FACT-8D Index score was lower than either the EQ-5D Crosswalk or EVS scores (0.66 vs 0.73 and 0.80, respectively).

**Table 1:** Baseline demographic characteristics and EQ-5D and FACT-8D Index scores for patients included for analysis (*n* = 250)

Variable	
**Age (years)**	
Mean (SD)	66.7 (9.3)
Median (IQR)	67 (61;73)
**Sex**	
Male	184 (73.6%)
Female	66 (26.4%)
**Race**	
White	215 (86.0%)
Asian	21 (8.4%)
Other	6 (2.4%)
Not reported or unknown	8 (3.2%)
**Region**	
Europe	200 (80.0%)
Asia	20 (8.0%)
Other	30 (12.0%)
**FACT-8D scores**	
Mean (SD)	0.66 (0.21)
Median (IQR)	0.71 (0.53;0.82)
**EQ-5D-5L Crosswalk scores**	
Mean (SD)	0.73 (0.23)
Median (IQR)	0.77 (0.64;0.88)
**EQ-5D-5L England value set**	
Mean (SD)	0.80 (0.20)
Median (IQR)	0.86 (0.74;0.94)

*SD* standard deviation; IQR: interquartile range

On FACT-8D at baseline, problems were most frequently reported on the work (87% reporting problems) and sleep (80%) dimensions, and least frequently on the nausea dimension (17%). 71.8% of patients reported some degree of fatigue. At 31 weeks, the largest reductions in reported problems were seen on the pain (15.2% fewer patients) and sadness (10.4% fewer) dimensions. At baseline, only 1.2% of patients reported no problems on any dimension.

On EQ-5D-5L at baseline, problems were most frequently reported on the pain/discomfort dimension (62% of patients reporting problems) and mobility (52%) dimensions, and least frequently on self-care (14%). At week 31, the largest reductions in reported problems were observed on the pain/discomfort and mobility dimensions, with almost 11% fewer patients reporting problems in both cases. At baseline, 16% of patients reported no problems on any dimension.

**Fig. 1:**
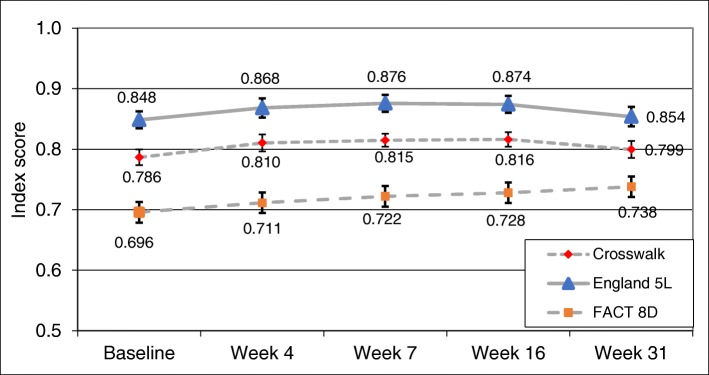
Mean FACT-8D and EQ-5D-5L (Crosswalk and England value set) Index scores and standard errors by study visit for patients completing the assessment at week 31 (*n* = 130)

Figure 1 shows the change in mean utility scores using only data from patients who were still on study at week 31 (*n* = 131). FACT-8D Index scores were lower than EQ-5D-5L scores over the period and showed a steady improvement from baseline through to week 31, though the mean change was small, at 0.042. In contrast, the EQ-5D-5L Index showed a small improvement to week 7 before plateauing and then returning to almost the original score by week 31 (mean change using the Crosswalk was 0.013).

### Convergent validity

The FACT-8D showed good convergent validity, with correlations following expected patterns. Pearson correlations between the FACT-8D and the EQ VAS, LymS, and HgB values were 0.50, 0.63, and 0.26, respectively. Correlations with other FACT summary measures were higher but are not reported here because of the overlap in content (FACT-8D items were drawn from the FACT-G). Results for EQ-5D-5L were similar, ranging from a correlation of 0.29 with HgB, to 0.50 with the EQ VAS, 0.60 with the LymS, and 0.70 with the FACT TOI. All correlations were statistically significant at *p* < 0.0001.

### Known groups’ validity

FACT-8D failed to discriminate between groups on four of the seven variables analysed, whereas EQ-5D-5L showed statistically significant differences between categories for all variables tested except prior lines of therapy, with the score differences in the expected direction (Table 2).

**Table 2:** Known groups’ validity for FACT-8D and EQ-5D-5L Index scores, using baseline data

	FACT-8D		EQ-5D-5L crosswalk		EQ-5D-5L EVS	
	Mean (SD)	***P*** value	Mean (SD)	***P*** value	Mean (SD)	***P*** value
**Baseline lymphoma symptoms**		**0.02**		**< .0001**		**< .0001**
No (*N* = 117)	0.69 (0.18)		0.81 (0.21)		0.87 (0.16)	
Yes (*N* = 133)	0.63 (0.28)		0.67 (0.22)		0.75 (0.21)	
**Presence lymphoma B symptoms**^**a**^		**< 0.01**		**< 0.001**		**< 0.001**
No symptoms (*N* = 186)	0.68 (0.20)		0.77 (0.20)		0.84 (0.16)	
One or more symptoms present (*N* = 64)	0.60 (0.32)		0.63 (0.28)		0.71 (0.26)	
**Other MCL-related symptoms**		0.10		**<.0001**		**< .0001**
No (*N* = 140)	0.67 (0.22)		0.79 (0.21)		0.85 (0.17)	
Yes (*N* = 110)	0.63 (0.27)		0.66 (0.24)		0.74 (0.22)	
**ECOG**^**b**^		**< .0001**		**< .0001**		**< .0001**
0 (*N* = 117)	0.74 (0.22)		0.82 (0.18)		0.88 (0.15)	
1(*N* = 130)	0.58 (0.25)		0.66 (0.23)		0.74 (0.21)	
**MIPI**		0.21		**< 0.001**		0.001
High Risk (*N* = 53)	0.61 (0.27)		0.63 (0.28)		0.72 (0.26)	
Intermediate (*N* = 118)	0.66 (0.24)		0.75 (0.18)		0.82 (0.16)	
Low Risk (*N* = 79)	0.68 (0.23)		0.78 (0.23)		0.84 (0.19)	
**HgB**		0.14		**< 0.001**		**< 0.001**
HGB < 130 g/L (*N* = 137)	0.60 (0.27)		0.69 (0.24)		0.77 (0.22)	
HGB > =130 g/L (*N* = 113)	0.64 (0.20)		0.79 (0.19)		0.85 (0.16)	
**Previous lines of therapy**		0.43		0.35		0.35
1 (*N* = 98)	0.68 (0.24)		0.74 (0.23)		0.81 (0.19)	
2 (*N* = 69)	0.64 (0.24)		0.75 (0.23)		0.82 (0.18)	
≥3 (*N* = 83)	0.64 (0.25)		0.70 (0.23)		0.78 (0.22)	

*ECOG* Eastern Cooperative Oncology Group, *EVS* England value set, *MCL* Mantle cell lymphoma, *MIPI* Mantle Cell Lymphoma International Prognostic Index, *HgB* haemoglobin

^a^Lymphoma B symptoms are symptoms common in lymphomas affecting B-cells and include, among others, fever, night sweats, persistent fatigue, or itchy skin. ^b^Patients in ECOG performance status 0 are patients considered to be fully active with no performance restriction. Patients in ECOG performance status 1 are those who are restricted in physically strenuous activity but ambulatory and able to carry out work of a light or sedentary nature

### Responsiveness

The FACT-8D showed good responsiveness, with statistically significant differences between score changes for patients categorised as improving, worsening or stable on the FACT-Lym total score, LymS, and HgB; all changes were in the expected direction (see Table 3). In patients classified as improving, effect sizes for the FACT-8D were slightly larger than those for the EQ-5D-5L, although results for the two instruments were quite similar when using the Cohen classification and the shared between FACT-Lym total score (which includes FACT-G) and the FACT-8D should be taken into account. Changes on the FACT-8D and EQ-5D-5L associated with change in ECOG status were non-significant, but there was relatively little change in ECOG status in general in the study.

**Table 3:** Results of responsiveness testing for FACT-8D and EQ-5D-5L using data from baseline to 31 weeks

	FACT-8D			EQ-5D-5L Crosswalk			EQ-5D-5L EVS		
	Mean change	P	ES	Mean change	P	ES	Mean change	P	ES
**FACT-Lym Total (6.5 point MID)** [23]		< 0.0001			<.0001			<.0001	
Improve (*N* = 51) ^a^	0.17		0.94	0.10		0.66	0.09		0.64
No change (*N* = 36)	0.01			−0.01			−0.01		
Worsen (*N* = 33)	−0.121		0.67	−0.11		0.73	−0.11		0.79
**LymS** **(5 point MID)** [24]		< 0.0001			<.0001			<.0001	
Improve (*N* = 46)	0.16		0.76	0.12		0.75	0.10		0.67
No change (*N* = 58)	−0.0002			−0.04			−0.04		
Worsen (*N* = 16)	−0.16		0.76	−0.12		0.75	−0.12		0.8
**ECOG status**		0.63	–		0.19	–		0.14	–
Improve (*N* = 26)	0.03			0.02			0.00		
No change (*N* = 82)	0.05			−0.02			0.02		
Worsen (N = 13)	−0.01			−0.07			−0.08		
**HgB, </> 120 (women) or 130 g/L (men)** [22]		0.002			0.02			0.005	
Improve (*N* = 15)	0.18		0.86	0.13		0.76	0.12		0.75
No change (*N* = 76)	0.05			0.01			0.00		
Worsen (*N* = 30)	−0.05		0.24	−0.03		0.18	−0.04		0.25

*ECOG* Eastern Cooperative Oncology Group, *ES* Effect size, *EVS* England value set, *HgB* Haemoglobin, *LymS* FACT-Lym lymphoma specific additional concerns subscale. ^a^The number of patients showing improvement/worsening/no change corresponds to the total number of patients still in the trial at Week 31 with available changes from baseline on each of the characteristics analysed

Correlations between change scores on the FACT-8D and other measures of health status, such as EQ-5D VAS, LymS, and haemoglobin, at week 31 were all moderate or strong (0.33, 0.54, and 0.24, respectively, *p* < 0.001), providing further evidence of responsiveness. Results were similar for EQ-5D-5L, though correlations with EQ VAS and the LymS were slightly higher (0.41 and 0.59, respectively, using Crosswalk values).

### EQ-5D and fatigue

The exploratory analysis to assess the sensitivity of the EQ-5D-5L to fatigue showed that both the EQ-5D-5L Crosswalk and EVS values were monotonically lower with increasing levels of severity of fatigue and lack of energy on the two corresponding FACT-Lym items. Differences in values across response categories were all statistically significant for both FACT items tested and for both value sets, suggesting good known groups’ validity for fatigue. The range of EQ Index scores between the lowest and highest levels of fatigue was considerable, for example, mean (SD) Crosswalk values for those reporting most problems on ‘tiring easily’ were 0.50 (0.28) and 0.87 (0.28) for those reporting no problems.

### Analysis by ECOG status

**Fig. 2:**
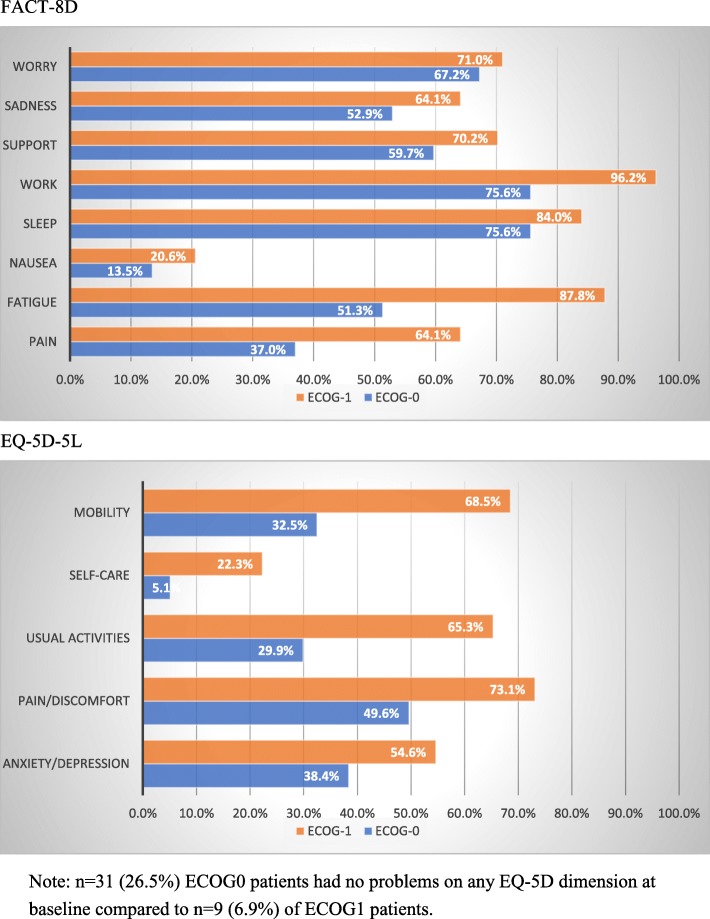
Frequency (%) of problems on FACT-8D and EQ-5D-5L dimensions at baseline, by baseline ECOG status. FACT-8D. EQ-5D-5L. Note: *n* = 31 (26.5%) ECOG0 patients had no problems on any EQ-5D dimension at baseline compared to *n* = 9 (6.9%) of ECOG1 patients

ECOG1 patients reported more problems than ECOG0 patients on all FACT-8D and EQ-5D dimensions (see Fig. 2). The differences were particularly noticeable on the FACT-8D dimensions of fatigue and pain and on the dimensions of Usual Activities and Mobility on the EQ-5D-5L. On EQ-5D-5L, 26.5% of ECOG0 patients reported full health (11111) at baseline, compared to 6.9% of ECOG1 patients.

**Fig. 3:**
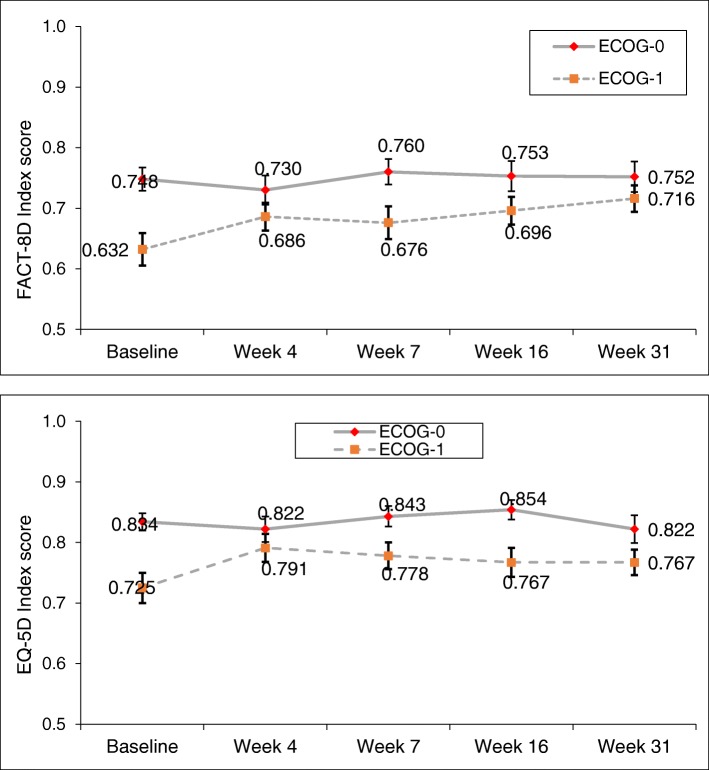
Mean FACT-8D and EQ-5D-5L Crosswalk utility scores and standard errors by study visit for patients who completed the assessment at week 31 (*n* = 76 for ECOG0, *n* = 54 for ECOG1), based on baseline ECOG status

Figure 3 shows mean FACT-8D and EQ-5D-5L crosswalk utility scores and their standard errors for patients who completed an assessment at week 31, according to their baseline ECOG status. On both Indices, values in the ECOG0 group were higher at baseline than those for ECOG1 patients and remained higher and relatively unchanged through to week 31. In the ECOG1 group, on the other hand, FACT-8D and EQ-5D-5L scores both showed considerable improvement over the same period (see Fig. 3), with FACT-8D Index scores increasing by 0.084 points, compared to an increase of only 0.004 points in ECOG0 patients (*p* = 0.024), and EQ-5D-5L Crosswalk Index scores increasing by 0.042 in the ECOG1 group, compared to a decrease of − 0.012 points in ECOG0 patients (*p* = 0.098). The rate of drop-outs was notably higher in the ECOG1 group.

## Discussion

This is the first study we are aware of to test the psychometric properties of the FACT-8D and compare them to the EQ-5D-5L. This type of comparison is critical in determining whether new, condition-specific PBMs such as the FACT-8D perform well in their target populations and whether they offer advantages over well-established generic measures such as EQ-5D.

The findings show that the FACT-8D has good convergent validity and responsiveness in this RR MCL trial population, although where analysis relied on correlation with or categorised change in FACT-Lym total scores, it should be noted that FACT-8D items were drawn from the FACT-G (which is included within FACT-Lym), which would inflate performance of the FACT-8D. Results for known groups’ validity were poorer, with the instrument failing to discriminate between groups classified by other MCL-related symptoms, MIPI score, HgB value, or prior lines of treatment.

The EQ-5D-5L, which is designed for use in a much wider range of populations, showed equally good convergent validity, similar levels of responsiveness, especially when considering Cohen’s classification, and outperformed FACT-8D in terms of known groups’ validity, only failing to discriminate between groups defined by number of prior lines of therapy.

It is not clear why the FACT-8D should perform less well than EQ-5D-5L in discriminating between groups in this study, although the coefficients used to model utilities may be a factor. In the Australian value-set, the same coefficients are attributed to different levels of severity in some dimensions. One such dimension is fatigue, where levels 1 and 2 are both assigned a coefficient of 0.00 and levels 4 and 5 a coefficient of 0.13, essentially reducing this to a three-level dimension. That might explain why the FACT-8D appeared to be less sensitive to differences in levels of HgB in known groups’ testing than the EQ-5D-5L, although both instruments proved responsive to changes in level of HgB. Identical coefficients for different severity levels are also found on the FACT-8D sleep, work, support, and worry dimensions which may reduce their capacity to discriminate between patients with different levels of health problems.

HgB was used as a proxy for fatigue in the current study. Additional analysis using results from the ‘tiring easily’ and ‘lacking energy’ items of the FACT-G indicated that the EQ-5D-5L Index scores (Crosswalk and EVS) discriminated across levels on both items. These results, coupled with those on HgB, suggest that EQ-5D-5L may capture the effects of fatigue quite well, in contrast to earlier claims to the contrary [8, 9], although level of fatigue experienced by this clinical trial sample is likely to have been limited by the relatively unimpaired performance status ECOG eligibility criteria. Other researchers have also shown that EQ-5D utility values differ between patients with and without anaemia [30].

Although this is the first study to compare the performance of EQ-5D-5L with the FACT-8D, other studies have compared the performance of EQ-5D with the EORTC-8D, a cancer-specific PBM scored from the EORTC QLQ-C30 questionnaire [9, 31, 32]. In their analysis of data from a large population-based cancer cohort, Lorgelly et al. [9] found that the two instruments showed high correlation but poor agreement. As in our study, van Dongen-Leunis et al. [31] found that PBM derived from the EORTC-QLQ30 and the EQ-5D-5L showed good convergent and known groups’ validity in acute leukemia patients, though the disease-specific PBM showed greater discriminatory power than EQ-5D-5L. In multiple myeloma patients, Rowen et al. [32] found that EORTC-8D utility estimates were broadly comparable to those obtained using EQ-5D, but that EORTC-8D better captured changes in HRQOL patients in mild health states.

A further finding of our study was that the EQ-5D-5L Crosswalk and EVS showed a similar pattern of change over time and a very similar performance in terms of their validity and responsiveness, suggesting that both are suitable for use in this population. The main difference was that the EVS produced consistently higher values than the Crosswalk and both EQ-5D indices produced higher values than the FACT-8D. While it would have been preferable to use Australian values with EQ-5D-5L to enhance the comparison with the FACT-8D, the EQ-5D-5L value set for Australia is currently unpublished and there are no EQ-5D-5L value sets available for comparable countries nearby, e.g. New Zealand. It should also be noted that a comparison of value sets across 7 countries, including the UK [33], found them to have similar characteristics, suggesting that the use of the EQ-5D-5L UK value set in this study is unlikely to have influenced the findings.

A novel aspect of the present analysis was the exploration of PRO outcomes by baseline ECOG status. We found that the ECOG0 group maintained relatively high utility scores throughout the study, with Crosswalk Index scores of 0.8 or more and substantial proportions of patients reporting ‘no problems’ on EQ-5D dimensions. Patients in ECOG0 in the present analysis therefore self-reported better health status than respondents of a similar age (> 65 years) in the general population of England [34], who reported a lower Index value (0.77) and higher levels of problems on all EQ-5D dimensions, except anxiety/depression. However, it should be noted that, while disease burden (i.e. short life expectancy) can be high in RR MCL, patients may have few symptoms and relatively unimpaired performance status. Furthermore, RR disease does not necessarily mean symptomatic disease, as indicators for relapse/progression could be clinical (chemistry and haematological counts).

On the other hand, the work by Tucker et al. [29] indicates that the proportion of ECOG0, high functioning RR MCL patients included in the RAY trial may be unrealistic in real-world clinical practice. The fact that we observed greater utility gains in those beginning the trial with somewhat limited performance status (ECOG1) compared to those with no performance limitation (ECOG0) at baseline might suggest that, if patients managed in clinical practice tend to be in poorer health than those in the RAY trial (e.g. ECOG2+), utility gains in clinical practice would be greater than those observed in the trial.

A limitation of this study was that we could only analyse data from RR MCL patients with ECOG0–1 performance status at baseline. Results may therefore not be generalisable to other types of MCL patients, for example, those with more severe disease or those who are not refractory or relapsed. We also had to use an Australian value set to calculate utilities for the FACT-8D as no other value set was available. Results could vary if other value sets become available in the future. Finally, there was substantial drop-out in the RAY trial which meant that utility values were available at follow-up visits for increasingly small samples. The likelihood that drop-out was due to disease progression or death i.e. those in poorer health states, may inflate mean utility values for later time-points. Due to study drop-out concerns, the time period for analysis was restricted to 31 weeks and mean utilities shown in Fig. 1 were only plotted for those still in the trial at week 31. Study drop-out will also have limited testing of responsiveness, particularly for decline. However, as indicated in Dreyling et al. (2016), by week 31 of the trial, over 40% of temsirolimus patients and approximately 70% of ibrutinib patients had shown an improvement in clinical symptoms by week 31 of the trial, while approximately 65% and 25%, respectively, had shown worsening at that point, suggesting that, from a clinical point of view, this was a reasonable time point to use for the responsiveness analysis.

## Conclusions

In conclusion, the FACT-8D provides an alternative system for obtaining utility values in cancer patients but, using the current Australian value set, it did not perform better than EQ-5D-5L in this RR MCL dataset, despite having a lower baseline ceiling effect, and was inferior in terms of known groups’ validity. Further testing of the FACT-8D is needed in other cancer populations and settings. The high baseline performance status in this RR MCL trial sample may mean that utility gains estimated using RAY trial data are likely to be smaller than those which would be seen in clinical practice.

## Data Availability

Data cannot be shared further due to ethical and proprietary considerations. Reasonable requests for analysis and aggregated results materials will be considered.

## Abbreviations

ANCOVA: Analysis of covariance
DCE: Discrete choice experiment
ECOG: Eastern Cooperative Oncology Group
EORTC: European Organisation for the Research and Treatment of Cancer
EVS: English value set
FACT: Functional Assessment of Chronic illness Therapy
FACT-G: Functional Assessment of Chronic illness Therapy - General
HgB: Haemoglobin
HRQOL: Health-related quality of life
LymS: FACT-Lym lymphoma specific additional concerns subscale
MID: Minimal important difference
MIPI: Mantle Cell Lymphoma International Prognostic Index
PBM: Preference-based measure
PRO: Patient reported outcome
RR MCL: Relapsed/refractory mantle cell lymphoma
SD: Standard deviation
TOI: Trial Outcome Index
TTO: Time trade-off
VAS: Visual analogue scale
